# Use of Yeast Cell Wall Extract for Growing Pigs Consuming Feed Contaminated with Mycotoxins below or above Regulatory Guidelines: A Meta-Analysis with Meta-Regression

**DOI:** 10.3390/toxins15100596

**Published:** 2023-10-03

**Authors:** Alexandra C. Weaver, Daniel M. Weaver, Nicholas Adams, Alexandros Yiannikouris

**Affiliations:** 1 Alltech, Inc., 3031 Catnip Hill Road, Nicholasville, KY 40356, USA; ayiannikouris@alltech.com; 2 Independent Researcher, Orrington, ME 04474, USA; dmw1121@gmail.com; 3 Alltech United Kingdom, Stamford PE91TZ, UK; nadams@alltech.com

**Keywords:** mycotoxins, meta-analysis, meta-regression, performance, pigs, yeast cell wall extract

## Abstract

Using a random-effects meta-analysis, the performance of growing pigs under a mycotoxin challenge (MT) with or without supplementation of yeast cell wall extract (YCWE, Mycosorb^®^, Alltech Inc.) was evaluated. Both MT and YCWE were also compared to animal controls not receiving mycotoxins (CTRL). Meta-regression was used to further explore the impacts of MT at/below (category 1) or above (category 2) global regulatory guidelines. Following the screening, 23 suitable references (30 mycotoxin treatments) were used. Overall, MT lowered average daily gain (ADG, *p* < 0.001) and average daily feed intake (ADFI, *p* < 0.0001) from CTRL by −84 and −165 g, respectively. Inclusion of YCWE during mycotoxin challenges (YCWE+MT, average 2.1 kg/ton) tended to result in greater ADG (+17 g, *p* = 0.068) compared to MT treatments. The gain-to-feed ratio (G:F) was not impacted by MT or YCWE+MT. Further investigation by meta-regression revealed that pigs fed MT in category 1 had lower ADG (−78.5 g, *p* < 0.001) versus CTRL, while YCWE+MT had higher ADG (+48 g, *p* < 0.001) over MT and was similar to CTRL. The ADFI was not impacted, although YCWE+MT had ADFI values similar to the CTRL. In category 2, ADG and ADFI of pigs fed MT were lower than CTRL (−85.1 and −166 g, respectively, *p* < 0.0001), with a tendency for YCWE+MT to result in higher ADFI (+25.3 g, *p* = 0.062). In summary, the inclusion of YCWE provided benefits to performance during common mycotoxin challenge levels (at or below regulatory guidelines).

## 1. Introduction

Mycotoxins are common contaminants of feedstuffs consumed by swine. Some of the well-known mycotoxins that are harmful to pigs include aflatoxin B1 (AFB1), ochratoxin A (OTA), deoxynivalenol (DON), T2/HT2-toxins, zearalenone (ZEA), and fumonisins (FUM) [1], but numerous additional mycotoxins are now being identified that may be frequent contaminants of feedstuffs. A recent survey conducted in the United States indicated that 98.6% of corn grain analyzed over a seven-year period contained mycotoxins, with an average of 4.8 mycotoxins per sample based on the 35 different mycotoxins investigated [2]. The most frequently detected mycotoxin in more than 78% of samples was fusaric acid (FA). Additionally, over 65% of samples contained DON and fumonisin B1 (FB1). Not only are mycotoxins a current threat to feed quality and safety, but they will likely continue to play an important role in the future. It is suggested that rising global temperatures and CO_2_ concentrations will have a significant impact on toxigenic fungi and the production of mycotoxins [3]. As such, swine producers may continue to experience an equal or greater number of mycotoxins, as well as higher concentrations of mycotoxins, in future feedstuffs and ration.

As the exposure to mycotoxins in swine feed increases, negative impacts on the gastrointestinal tract, internal organs, reproductive system, and immune system can be expected [4]. These effects, in turn, lower the growth performance and health of pigs and the profitability of swine operations even under chronic exposure. Although it may be easier to observe changes to health or performance at higher concentrations of mycotoxin exposure, i.e., those above European Union (EU) regulatory guidelines for swine feed of 0.005 to 0.01 mg/kg AFB1, 0.05 mg/kg OTA, 0.9 mg/kg DON, 5 mg/kg FUM, and 0.1 to 0.25 mg/kg ZEA [5,6,7], there is a growing body of literature showing that lower or chronic levels of mycotoxins also have negative effects. At concentrations below EU guidelines, DON and FUM are both shown to alter intestinal microbial populations, leading to an increase in pathogenic microorganisms and reduced microbial diversity [8,9]. Furthermore, combining lower levels of mycotoxins together can increase the overall negative response in the pig, leading to a greater reduction in feed intake, body weight loss, an increase in expression of pro-inflammatory cytokines, and an increase of pathogenic microorganisms in the intestinal tract [10].

To minimize the risk of mycotoxins to swine, it is important to have a proper management program in place on-farm that includes testing, monitoring, and mitigation [11]. One of the best ways to directly protect the animal from mycotoxins is the use of feed additives or ingredients added to rations [12]. A proposed mechanism is through binding mycotoxins in the gastrointestinal tract to prevent absorption and thus minimize the negative effects of mycotoxins in the animal. Among these products, the yeast cell wall extract (YCWE; Mycosorb^®^, Alltech, Inc., Nicholasville, KY, USA) rich in complex insoluble carbohydrates demonstrates considerable ability for the binding of several mycotoxins in vitro, ex vivo, and in vivo [13,14,15,16]. Although numerous papers have been published that investigate the use of YCWE in pigs exposed to mycotoxins, it may be difficult to make an overall conclusion on the use of YCWE due to variations between individual study responses and to the limited number of animals subjected to testing. To further understand the use of YCWE in swine diets containing mycotoxins, a random-effects meta-analysis with meta-regression was conducted that allows for integration and quantification of an overall outcome across the body of scientific work related to the use of YCWE.

Meta-analysis output can provide a more precise estimate of treatment effects than an individual trial owing to the statistical evaluation of a wider data pool [17]. The use of the random-effects model incorporates an estimate of between-study variation or heterogeneity [18]. Heterogeneity is observed when intervention effects vary more than expected by random chance [17]. Heterogeneity in mean effect sizes among studies may be due to differences in experimental design and methodology, including differences in target populations, analytical methods, measurement instruments, doses of interventions, and timings [19,20]. When heterogeneity is high (*I^2^* statistic approaches a maximum of 100%), variability could exist, and results may be interpreted with caution [17,20]. However, through further assessment by meta-regression, a portion of the heterogeneity may be explained. In this current research, the selected database was also assessed by meta-regression to investigate how mycotoxin concentrations play a role in the effects on pigs and the response to a YCWE mitigation use during mycotoxin challenges. There were two objectives of this study. The first objective was to conduct a meta-analysis to assess the impact of mycotoxin-contaminated feed (MT) at all levels on the measured incidence of weight gain, feed intake, and efficiency of pigs in contrast to pigs fed control diets without mycotoxins (CTRL) or pigs fed YCWE during mycotoxin challenges (YCWE+MT), as well as determine if pigs fed YCWE+MT had returned performance back to CTRL. The second objective was to assess the same three treatment comparisons categorically by meta-regression: all mycotoxins at/below EU and USA regulatory guidelines (category 1) and at least one mycotoxin above regulatory guidelines (category 2).

## 2. Results

### 2.1. Research Characteristics

A total of 23 references (14 were conducted with nursery pigs and 11 with grow-finish pigs), which contained 30 different mycotoxin levels, were used for this meta-analysis (Table 1). The studies were published over a 20-year period (2002 to 2022) and were conducted in 10 different countries (six from the USA, five from Finland, four from Canada, two from Germany; one each from Belgium, China, Italy, Lithuania, Republic of Korea and Serbia). There were 3165 pigs included: 516 fed CTRL, 1293 fed MT, and 1356 fed YCWE+MT. Trials were conducted over a length of 14 to 115 days (median 26 days; mean 36.2 days). Most trials had diets naturally contaminated with mycotoxins (Table 1), but six used pure mycotoxin sources. Mycotoxin types reported by these studies in the MT rations (Table 1) included aflatoxins(AFs)/AFB1 (8 trials), OTA (1 trial), DON (16 trials), 15-acetyl-deoxynivalenol (2 trials), T-2 toxin (1 trial), FUM (6 trials), ZEA (15 trials), and FA (2 trials). The overall average dose rate of YCWE was 2.1 kg/t (Table 1), with further breakout by category resulting in an average YCWE inclusion rate of 2.25 kg/t (range 1.0 to 4.0 kg/t) for trials in category 1 and 2.11 kg/t (range 0.5 to 4.0 kg/t) for category 2.

### 2.2. Meta-Analysis: Overall Impact on Pig Performance

The consumption of MT diets by growing pigs resulted in significantly lower (*p* < 0.001) average daily gain (ADG) than pigs fed control diets, with a mean difference of −84 g (Table 2). The feeding of YCWE during mycotoxin challenges tended (*p* = 0.068) to result in higher ADG by 17 g over MT. The ADFI of pigs fed MT was significantly lower (*p* < 0.0001) by −165 g compared to CTRL (Table 2), while pigs fed YCWE did not differ from MT. Although ADG and ADFI were impacted, the consumption of MT or YCWE by pigs did not significantly alter the overall mean effect for G:F (Table 2). Forest plots are provided in the Supplementary Materials section, Figures S2–S4.

Between-study heterogeneity and publication bias were assessed (Table 2). The results for heterogeneity (I^2^) showed significant differences (*p* < 0.001) between studies for all treatment comparisons and parameters tested. The I^2^ values were above 57.66% for all comparisons except for ADFI between YCWE and MT. Eggar’s test for asymmetry did not indicate publication bias (*p* > 0.05) for all treatment effects of ADG, ADFI, and G:F (Table 2 and Supplementary Materials Figures S5–S7).

### 2.3. Meta-Regression: Mycotoxin Impacts below or above Regulatory Guidelines

**Figure 1:**
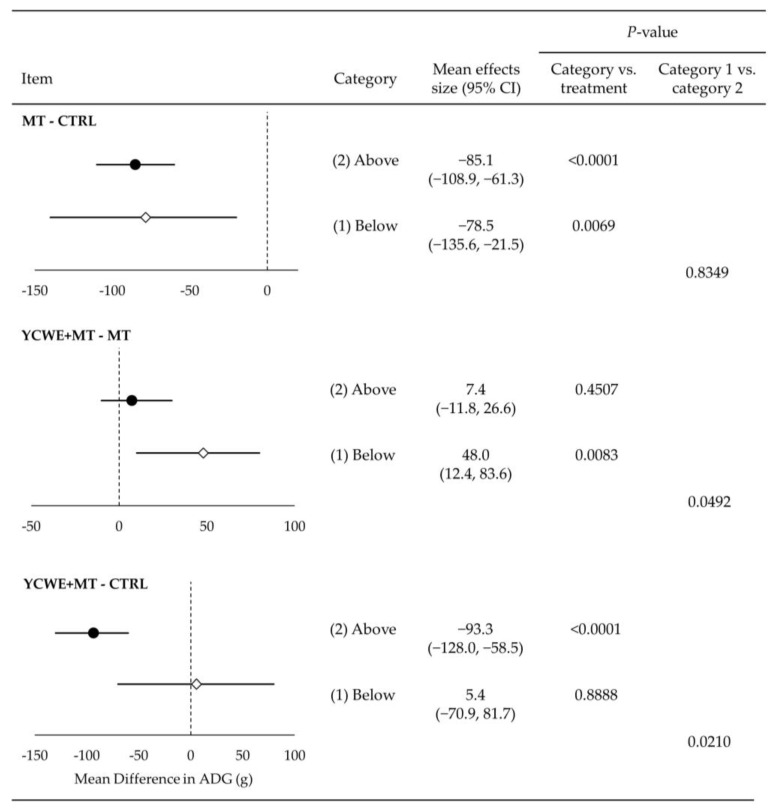
Mean effect size estimates with 95% confidence interval (95% CI) for average daily gain (ADG) in grams for growing pigs from the mixed-effect meta-regression between treatments of mycotoxins and control (MT-CTRL), yeast cell wall extract in the presence of mycotoxin challenges (YCWE, Mycosorb^®^, Alltech, Inc.) and mycotoxins alone (YCWE+MT-MT), and YCWE and control (YCWE-CTRL). Each trial used in this meta-regression was placed into one of two categories, which included (1) at/below (white diamonds) or (2) above (black circles) EU and US regulatory guidelines for mycotoxins in swine feed.

**Figure 2:**
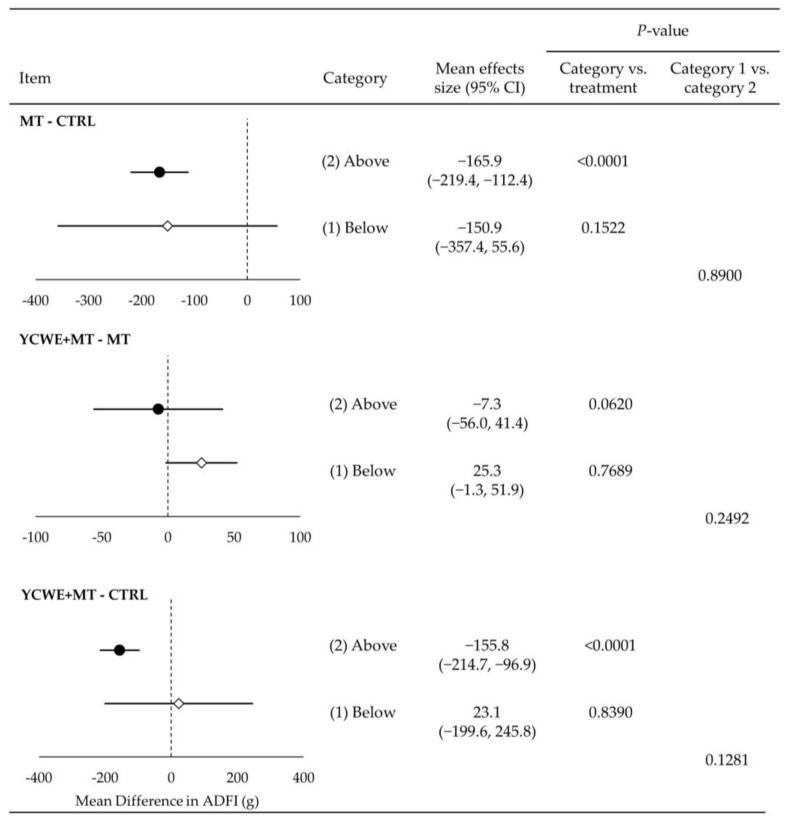
Mean effect size estimates for average daily feed intake (ADFI) in grams for growing pigs from the mixed-effect meta-regression between treatments of mycotoxins and control (MT-CTRL), yeast cell wall extract in the presence of mycotoxin challenges (YCWE, Mycosorb, Alltech, Inc.) and mycotoxins alone (YCWE+MT-MT), and YCWE and control (YCWE-CTRL). Each trial used in this meta-regression was placed into one of two categories, which included (1) at/below (white diamonds) or (2) above (black circles) EU and US regulatory guidelines for mycotoxins in swine feed.

**Figure 3:**
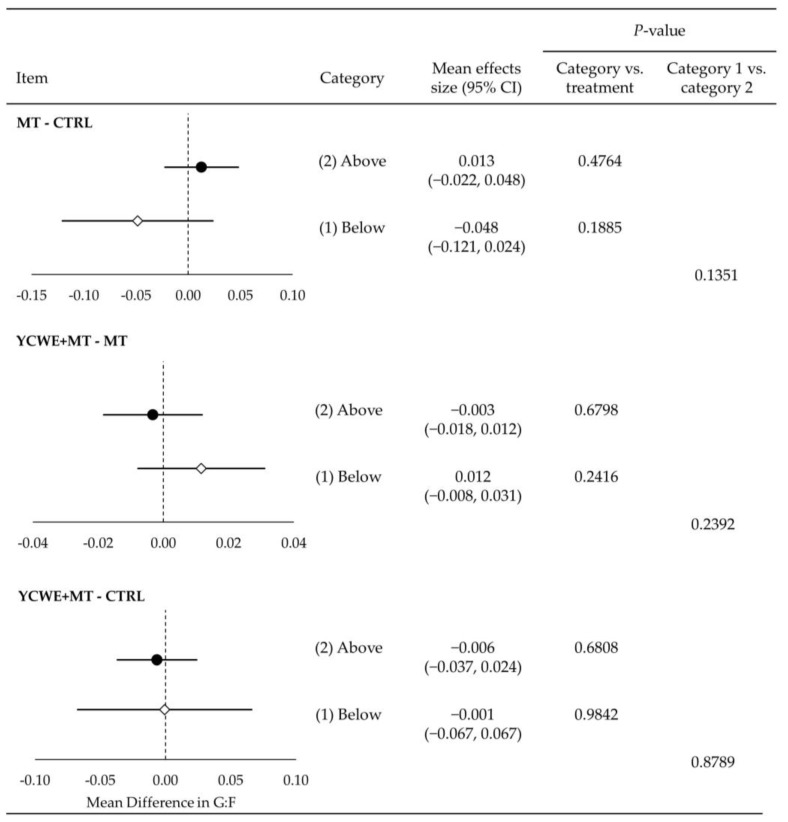
Mean effect size estimates for gain-to-feed ratio (G:F) for growing pigs from the mixed-effect meta-regression between treatments of mycotoxins and control (MT-CTRL), yeast cell wall extract in the presence of mycotoxin challenges (YCWE, Mycosorb, Alltech, Inc.) and mycotoxins alone (YCWE+MT-MT), and YCWE and control (YCWE-CTRL). Each trial used in this meta-regression was placed into one of two categories, which included (1) at/below (white diamonds) or (2) above (black circles) EU and US regulatory guidelines for mycotoxins in swine feed.

Growing pigs fed MT in category 1 had a mean difference of −78.5 g lower ADG (Figure 1) than those fed CTRL (*p* < 0.001). At the same time, the inclusion of YCWE resulted in higher (*p* < 0.01) ADG by 48.0 g over MT, which was similar to the CTRL (*p* = 0.888). Although ADG was impacted in category 1 trials, ADFI and G:F were not altered by MT or YCWE (Figure 2 and Figure 3).

Assessment of category 2 treatments indicated that pigs fed MT had lower ADG (Figure 1) from CTRL by a mean difference of −85.1 g (*p* < 0.0001). This reduction in ADG was not different from the loss in gain observed in category 1. The inclusion of YCWE in category 2 did not result in a significantly different ADG from MT, although it was numerically higher, and there was a difference between category 1 and 2 results for YCWE inclusion (*p* < 0.05). The ADFI (Figure 2) of pigs fed MT in category 2 was lowered (*p* < 0.0001) by −166 g, which was not different from category 1. Feeding of YCWE tended (*p* = 0.062) to result in higher ADFI over MT alone by 25.3 g, although it was not fully returned to that of CTRL. The G:F was not altered by MT or YCWE in category 2 trials (Figure 3).

## 3. Discussion

The current meta-analysis with meta-regression focused on the use of YCWE during mycotoxin challenges as a method of mycotoxin management for swine. Using data from 23 references (30 different mycotoxin treatments), this research investigated the benefits of using this adsorbent to support growth performance. Results indicated that mycotoxins of different types and concentrations can have an overall impact on the performance of growing pigs, resulting in significantly reduced ADG by −84.0 g and ADFI by −165.0 g. A previously published meta-analysis, which included a database of 85 published papers, also indicated that mycotoxins play a role in the gain and feed intake of pigs [44]. In that study, the data showed that mixtures of mycotoxins significantly lowered daily gain by −135 g and daily feed intake by −270 g. These effects are greater than we reported but within a similar amplitude, considering differences in mycotoxin type and content between the two meta-analyses. In contrast, another meta-analysis reported the effects of mycotoxins on pig ADG to be more similar to our results, with a mean difference of −80 g/d gain for research where pigs consumed DON in contrast to −69 g/d for FUM [45]. For any of these trials, slight differences could arise due to the mycotoxins present and the potential interactions between mycotoxin [46,47,48]; various forms [49,50] including conjugated forms [51] or unaccounted mycotoxins, such as emerging toxins [52,53,54].

The effect of mycotoxins on pig feed efficiency may be controversial, as conflicting results are reported. Unlike the results in this current work that showed no impact on efficiency, Holanda and Kim [16] reported that DON and FUM increased G:F, ZEA lowered G:F, and aflatoxins (AFs) had no influence. However, in other examples, mycotoxins had opposite effects, where AFs, DON, and FUM lowered G:F, and ZEA had no impact [44,45]. These variable effects on efficiency appear to depend not only on mycotoxin type but also concentration as well as other factors such as pig age and sex [44]. The lack of significance for G:F in the current meta-analysis may be due to those differences and/or a combination of effects.

Mycotoxin detoxifying agents can be added to the feed to counteract the impacts of mycotoxins on animals. Those products vary in composition and modes of action [16,55,56,57]). The YCWE studied herein contains a combination of insoluble carbohydrates associated with a complex parietal network, which have shown adsorptive properties toward a variety of mycotoxins, including AFB1, ZEA, DON, and FUM, and maintain this adsorption throughout pH changes of the intestinal tract [14,16]. Specifically, β-D-glucans in the yeast cell wall are largely responsible for the interaction with mycotoxins by van der Waals forces and weak hydrogen bonding. Once bound to YCWE, mycotoxins are less likely to exert negative effects on the animal’s immune, intestinal, endocrine, and internal organ systems. A previously published meta-analysis with broiler chickens showed the benefits of using YCWE during mycotoxin challenges with higher gain and feed intake, lower feed conversion ratio, and less mortality [58]. In the current meta-analysis with swine, results also showed the tendency for an overall greater ADG based on combined trial comparisons at all mycotoxin levels. Although support from broiler research is helpful, pigs may have a different response to mycotoxins and mitigation strategies. Due to a variety of factors, including the absence of pre-intestinal microbial transformation of some mycotoxins and an increase in affinity of cellular receptors towards others, swine are generally considered to have greater sensitivity to mycotoxins, causing them to be one of the more impacted animals [59] even at low doses [60,61,62].

Although it can be interesting to see the overall impact of mycotoxins on pig performance, perhaps more helpful are the results from the meta-regression investigating the effect of mycotoxin contamination level as the covariate. Trials with all reported mycotoxins at or below EU and USA regulatory advisory levels were placed into category 1, while those with at least one mycotoxin above advisory levels were placed into category 2. This covariate (mycotoxin level) was applied to compare the effects on growth performance without or with YCWE at challenge levels that were either lower and thus more likely to be experienced by pigs on a daily basis in common field conditions (i.e., category 1) versus higher levels of mycotoxins which may be consumed more sporadically (i.e., category 2). Accommodating more covariates would decrease the sample size between categories, increase variance, and be uninformative in identifying causal relationships. Additionally, low sample sizes between categories and repeated analyses with different covariates may increase the likelihood of making type 1 or type 2 errors and reduce the ability to identify causal relationships. Furthermore, previous literature has already assessed some other covariates, such as mycotoxin type, on pig growth performance [45]. Although the information on mycotoxin type can be helpful, pigs may more often be exposed to multiple mycotoxins rather than single mycotoxins [2]. As such, results from the current meta-analysis with meta-regression investigating mycotoxin levels either below or above regulatory guidelines could represent an original scientific approach and a novel understanding of real-world effects experienced on farms from multiple mycotoxins and chronic challenges.

It could be expected that mycotoxin levels in category 2 will impact swine performance; for example, 2000 μg/kg DON reduced the growth rate of gilts, increasing the time to reach 110 kg by 14.1 days [47]. However, the current research provides support that mycotoxins at or below advisory levels suggested by the EU and USA can still impact pigs’ performances. Concentrations below regulatory guidance are frequently detected [2,63,64,65] and could induce further strain on economic animal production systems [66,67]. This consideration is often absent from the regulatory mandate that exclusively focuses on feed safety and animal health. For example, the results of this meta-analysis showed that pigs fed MT had lower ADG than those fed CTRL by −78.5 g. Similarly, studies feeding pigs a mixture of DON and ZEA close to EU regulatory guidelines reported a loss of about −46 g/day in gain [10]. The focus of the present meta-analysis was on how growth performance can be altered by lower mycotoxin levels, but it is important not to forget about other health parameters. Previously published research has shown that the immunity of pigs may be impacted by 50 μg/kg OTA, as seen by an increase in the expression of pro-inflammatory cytokines [68]. Gut health is an initial target of many mycotoxins. For example, both DON and ergot alkaloids at levels close to EU regulatory limits reduced intestinal villus height [69,70], which could impact digestive health. Feeding pigs 800 μg/kg DON reduced the diversity and abundance of microorganisms in the colon [8], while DON at 750 μg/kg during challenges with Porcine Epidemic Diarrhea Virus increased the diarrhea rate [69]. These effects on gut health could impact energy and nutrient absorption, morbidity and mortality, and disrupt homeostasis [71,72]. Interestingly, and in contrast to these mentioned negative effects on gut health and function, lower levels of T-2 toxin at 15 or 83 μg/kg are implicated in reducing levels of *Salmonella typhimurium* in the bowel contents of pigs [73]. Despite this effect, pigs fed 83 μg/kg still had reduced weight gain. Lower levels of mycotoxins may also be implicated in physical damage, such as tail necrosis. A case study by van Limbergen et al. [74] reported a high prevalence of DON in feed samples of swine herds with high rates of tail necrosis. The average DON level was 484 ± 212 μg/kg on farms with clinical tail necrosis. In this report, pigs consuming these chronic DON concentrations also had fewer piglets weaned per sow per year [74].

Regarding the use of YCWE, this research was able to acknowledge additional beneficial properties by completing a meta-regression that took into account a categorial classification approach of mycotoxin concentrations using regulatory guidelines’ thresholds. During challenges with lower mycotoxin levels, fitting the category 1 definition, pigs fed YCWE+MT had significantly greater ADG by a mean difference of 48.0 g, equaling that of the CTRL-fed pigs. Mycotoxin concentrations in category 1 trials were at or below regulatory advisory limits and thus were at levels that swine producers may experience on a daily basis. A survey conducted between 2013 and 2019 showed that 75% of USA corn grain samples collected at harvest had DON ≤ 914 μg/kg and FB1 ≤ 4750 μg/kg [2]. As such, during these common mycotoxin challenges, the use of YCWE may be an effective method to regain optimal growth performance. Furthermore, as mycotoxin levels increased (category 2 trials), the ADG and ADFI continued to be numerically better using YCWE. There was a large variation in mycotoxin levels for category 2 trials, specifically DON up to 6100 μg/kg, FUM up to 14,000 μg/kg, and ZEA up to 1300 μg/kg, with additional high levels of FA reported in certain trials. As such, category 2 had a wider range of mycotoxin concentrations than category 1 and could be the cause of the observed reduced significance (although still numerically better) when using YCWE for category 2 challenges.

Improvement in growth performance is most likely the result that nutritionists and swine producers will focus on when considering using a mycotoxin management solution. However, YCWE use during mycotoxin challenges could have several other benefits for swine health, which were not assessed in our meta-analysis due to a limited number of available trials but could be important on-farm. For example, in diets naturally contaminated with *Fusarium* mycotoxins, YCWE indirectly helped maintain the brain neurochemistry of the pig through a limited increase in brain serotonin and hydroxyindolacetic acid, which in turn prevented a *Fusarium*-associated reduction in feed intake and lethargies [75]. Furthermore, pigs consuming YCWE during mycotoxin challenges were shown to have lower oxidative stress levels [33,76], stronger immunoglobulin status [33,39], and fewer negative impacts of mycotoxins on intestinal tract morphology [13,41]. Additionally, research that investigated the gut microbiome indicated that YCWE could play a role in reducing pathogenic bacteria while preserving commensal microbial diversity in the gastrointestinal tract across various animal and fish species [33,77].

## 4. Conclusions

Results from this random-effects meta-analysis with meta-regression showed that the consumption of mycotoxin-contaminated feed at levels both at/below and above regulatory guidance limits could negatively impact the growth performance of pigs. Importantly, this meta-analysis also provides useful information to swine nutritionists and producers on managing mycotoxins through the use of YCWE, with particular support for its use when mycotoxins are at levels commonly experienced out in the field. In this case, not only were pig performances enhanced and improved over the mycotoxin challenge alone, but they were also returned to that of the pigs not consuming mycotoxins. As such, the use of YCWE in rations at an average rate of about 2.0 kg/ton could be recommended to restore optimal levels of performance and profitability in swine operations.

## 5. Materials and Methods

### 5.1. Literature Search and Selection of References

A literature search was conducted in April 2022. Both published studies and unpublished trial reports that evaluated the effect of YCWE (Mycosorb^®^, Alltech Inc., KY, USA) inclusion during mycotoxin challenges on the impact of growing pig performance were used in this meta-analysis. The cell wall extract from YCWE composes 15–30% of the dry weight of the *Saccharomyces cerevisiae* cell used, with the major components being β-(1,3)-D-glucans and β-(1,6)-D-glucans (defined as glucans, which have been correlated to the adsorption activity to mycotoxins and represented between 25 and 35% of the yeast cell wall extract mass), mannoproteins (contributing to no less than 18% of the protein of the cell wall) and chitin. These components are covalently linked into macromolecular complexes, which are assembled to form the intact wall, often described as a ‘flexible building block’ [78,79] forming a three-dimensional network surrounding the entire cell. This network is held together by local alignments between segments of β-(1,3)-D-glucan molecules, allowing the formation of multiple hydrogen bridges. Clay material is present in the tested products as an anticaking agent, not to exceed a 2% inclusion rate within the material.

Published literature search was conducted through online databases (Google Scholar, Agricola, Pubmed). Search keywords included “mycotoxins” and “pigs” or “swine” in all searches, along with at least one additional keyword of “yeast cell wall extract”, “esterified glucomannan”, “glucomannan polymer”, “Mycosorb^®^”, or “Alltech”. There were no date or region restrictions placed on the search engines. Additionally, available unpublished trials from either conference presentations or Alltech’s internal database were included in this meta-analysis to provide a broader range of data. Increasing the number and sources of data, including unpublished data, may reduce the chance of publication bias [80,81].

Following the literature search, both published and unpublished research was subject to selection screening based on the following conditions: (1) trials were conducted with either nursery pigs or grow-finish pigs; (2) research contained at least one MT treatment as well as at least one treatment with YCWE+MT; (3) the inclusion of a control treatment without detectable mycotoxins or minimal mycotoxin content (CTRL) was desired but not required; (4) mycotoxin type and concentration details were required; (5) YCWE inclusion rate was required; (6) at least one variable of performance including ADG, ADFI, and G:F were provided; and (7) the trial reported the number of days in the experimental period and the number of animals. A PRISMA flow diagram showing the systematic review process is shown in the Supplementary Materials section, Figure S1, following the data collection process described by Page et al. [82].

To further assess the impact of different mycotoxin levels on pig performance, meta-regression methods were applied to categorize each trial into either category (1) all reported mycotoxins at or below regulatory advisory limits of the EU and US for swine finished feed (Table 3); or category (2) at least one reported mycotoxin above these limits. A combination of regulatory levels was utilized for category cutoff levels; specifically, EU limits were used for all mycotoxins listed in Table 3 other than DON. The United States Food and Drug Administration advisory guidelines for DON of 1000 μg/kg [83] were used rather than the EU guidance of 900 μg/kg [5] in order to include two additional trials into category 1 (trials [24] and [27]) for overall better distribution of category trials. These two trials had DON levels at 990 μg/kg [24] and 1000 μg/kg [27]. Appropriate limits were used for the age of pigs in each trial, either nursery or finishing, if different guidelines were specified for that mycotoxin type. There are no published limits for total type B trichothecenes; thus, the guidelines for DON were used in this case. Furthermore, FA was present in several trials. There are no regulatory guidance levels for FA, so it was not factored into the category breakdown, but all trials with FA were in category 2 due to higher concentrations of other mycotoxins.

### 5.2. Data Extraction

The impact of MT and YCWE+MT treatments on pigs was assessed through the performance variables of ADG, ADFI, and G:F. The average daily gain was provided in all but two trials, with the latter providing total weight gain. In these two cases, we calculated ADG using the published total gain divided by the number of trial days. There were four sources that did not provide feed intake information, but all others supplied ADFI. Most references provided G:F, but there were five that provided feed conversion ratio (i.e., feed to gain). In these cases, G:F was calculated manually from the results for ADG and ADFI. Additionally, one trial did not provide information on feed efficiency or ADFI; thus, only ADG was used for this trial.

### 5.3. Statistical Analysis

#### 5.3.1. Meta-Analysis

Mean effect sizes were calculated from raw mean differences of each response variable (i.e., ADG, ADFI, or G:F) from the treatment groups (CTRL, MT, and YCWE+MT) reported in each study. The standard error (SEM) and sample size for each treatment group were also collected from each trial. Where SEM was not reported, an average SEM for each treatment group from available studies was computed, which served as a value for missing data.

A hierarchical random-effects meta-analysis was employed as a first step of analysis [58,84,85], which assumed a priori heterogeneity in mean effect sizes among studies due to differences in experimental design and methodology [19]. A meta-analysis is a quantitative, formal, statistical method used to systematically assess and integrate the results of a body of research in order to derive conclusions [17]. Outcomes from the meta-analysis may generate a more precise estimate of treatment effects than any individual study as a result of the pooled data. The random-effects model incorporates an estimate of between-study variation or heterogeneity [18]. The meta-analysis model was specified as,

(1) $${\hat{\theta }}_{i}=\mu +e_{i}+\zeta_{i}\;$$

where $\hat{\theta }$_i_ is the observed effect size in the *i*-th study, *μ* represents an average true effect size from a distribution of true effect sizes, *e*_i_ is the associated sampling error with *e_i_*~*N* (0,*v*_i_), and ζ_i_ is the error associated with the distribution of effect sizes (i.e., between-study heterogeneity) with ζ_i_~*N* (0, τ^2^). Sampling variance (*v*_i_) was used from the studies or imputed using the method described above. The variance associated with the distribution of effect sizes (*τ*^2^) was estimated using restricted maximum likelihood [86]. A data transformation to assume normally distributed estimates was not used.

Funnel plots were generated to investigate publication bias. Funnel plots are scatter plots graphing the effect estimates from individual trials plotted against study precision [87]. Effect estimates for larger studies will be narrower, whereas smaller studies will scatter more widely at the bottom of the graph. Publication bias was quantified through the use of the Egger test [88]. The Egger test examines asymmetry in funnel plots of effect size and SEM and may suggest publication bias. Between-study heterogeneity was evaluated using the calculated heterogeneity statistic (*I*^2^) [89,90]. Heterogeneity is a measure of the diversity of effects between studies and may be detected if the variation between results is above that expected by chance [91]. This statistic is calculated as:

(2) $$I^{2}=\frac{Q-df}{Q}\times 100\%$$

where *Q* is the calculated chi-squared statistic and *df* is the degrees of freedom associated with a comparison. Calculated heterogeneity (*I*^2^) values represent unaccounted differences in mean effect sizes among the treatments (CTRL, MT, and YCWE+MT) for each response variable (ADG, ADFI, and G:F). Smaller values of *I*^2^ (<50%) indicate lower heterogeneity, while larger (>50%) values indicate higher heterogeneity [17,89]. Among all analyses, *I*^2^ values ranged from 26 to 92%. A similar wide range in *I*^2^ values is reported in other meta-analysis work [87]. It was hypothesized that dividing trials by mycotoxin content would explain additional unaccounted variability from the overall meta-analyses.

#### 5.3.2. Meta-Effects Meta-Regression

Unaccounted heterogeneity was explored by examining the effects of mycotoxin concentration, the covariate, on the overall meta-analysis using mixed-effects meta-regression. Each trial was placed into one of two categories, 1 or 2, described previously. These two categories were chosen as a way to assess the effects of mycotoxins at levels either below regulatory guidance and thus may be commonly experienced on-farm or above regulatory guidance, which may be experienced more sporadically. As such, to examine the effects of mycotoxin concentration on pig performance, a mixed-effects meta-regression was employed [87,92]. The model was specified as:

(3) $${\hat{\psi }}_{i}=\psi +\beta xi+e_{i}+\zeta_{i}\;$$

where $e_{i}\;\sim N\;\left(0,\;\nu_{i}\right)$ and $z_{i}\;\sim \;N\;(0,{\;\tau }^{2})$. The model estimates the mean effect size (${\hat{\psi }}_{i}$) in the *i*-th study as a function of the covariate mycotoxin level (*x_i_*) with regression coefficient *β*. The error terms *e*_i_ and ζ*_i_* were defined previously from our overall meta-analysis. Calculated meta-regression coefficients were used to describe linear relationships between mean effect sizes of treatment groups (CTRL, MT, and YCWE+MT) and response variables (ADG, ADFI, and G:F) for the two category levels. Two regressions that included or excluded the intercept term were evaluated. First, a test of the meta-regression without the intercept allowed examination of how the subgroups (categories 1 and 2) influenced the two mean effect sizes being compared and whether those effect sizes were statistically different from zero. Second, a test of the meta-regression with the intercept allowed for determining if there were statistical differences in mean effect size between the two categories of mycotoxin concentrations. The random-effects meta-analysis and meta-regression were conducted using R [93], R Studio (version 1.4.1106, RStudio, Boston, MA, USA) [94], and the package metafor (‘META-analysis FOr R’) [95]. Statistical significance was considered at an alpha value less than 0.05 and between 0.05 and 0.10 as the tendency.

## Figures and Tables

**Table 1:** Description of studies utilized for the random-effects meta-analysis examining the effects of mycotoxins without or with yeast cell wall extract inclusion on the performance of growing pigs.

								Reported Mycotoxins, μg/kg ^1^						
Ref.	Location	Pigs/trt ^2^	Ini. Wt., kg ^3^	Phase ^4^	Days ^5^	YCWE, kg/t ^6^	Contam. ^7^	AFs	OTA	DON, Type B	T2	FUM	ZEA	FA
Cat. 1 ^8^														
[21]	Italy	5	110.0	GF	28	2.0	Pure	20						
[21]	Italy	5	110.0	GF	28	2.0	Pure		50					
[22]	Lithuania	65	9.6	N	38	2.0	Natural	3.7		40			55	
[22]	Lithuania	65	27.4	GF	62	2.0	Natural	3.7		40			55	
[23]	Germany	12	-	GF	84	1.0	Mix			560			82	
[24]	USA	12	7.5	N	36	2.0	Natural	1		990		270	45	
[25]	Finland	8	35.2	GF	28	4.0	Pure	15					202	
[26]	Finland	4	35.2	GF	28	4.0	Natural			730			2	
[27]	USA	113	8.5	N	21	2.0, 3.0	Natural			1000				
[28]	Finland	6	36.0	GF	28	4.0	Pure						217	
[29]	Canada	337	7.3	N	39	1.0	Natural			700				
[30]	Belgium	5	5.7	N	18	1.0	Pure				83			
Cat. 2 ^8^														
[31]	Germany	20	7.7	N	35	2.0	Natural			4440				
[24]	USA	12	7.5	N	36	2.0	Natural	45		3450		620	96	
[32]	Finland	8	31.2	GF	28	4.0	Pure						268	
[33]	USA	30	55.6	GF	35	2.0	Natural	180				14,000		
[33]	USA	12	6.0	N	48	2.0	Natural	180		1000		9000		
[34]	South Korea	4	61.7	GF	14	4.0	Natural			2940				
[27]	USA	113	8.5	N	21	2.0, 3.0	Natural			3900				
[35]	Serbia	8	14.4	N	31	1.0	Natural						384	
[36]	USA	260	22.9	GF	115	1.0, 2.0	Natural			4870		670	570	
[37]	China	25	8.0	N	28	2.0	Natural			1990		290	650	
[37]	China	25	8.0	N	28	2.0	Natural			3980		580	1300	
[38]	USA	30	6.0	N	35	2.0	Natural	32				1600		
[39]	Canada	35	10.0	N	21	0.5, 1.0, 2.0	Natural			5100			300	23,400
[40]	Canada	30	9.3	N	21	2.0	Natural			3900			200	36,200
[40]	Canada	30	9.3	N	21	2.0	Natural			6100			500	49,300
[41]	Canada	10	6.0	N	14	1.0	Natural			4610				
[42]	USA	21	9.1	N	42	2.0	Natural			4800			300	
[43]	Finland	10	36.5	GF	28	4.0	Culture			1139			289	600

^1^ Mycotoxin types and levels reported in the studies used in this meta-analysis. AFs: aflatoxin B1 or total aflatoxins including B1 + B2 + G1 + G2; OTA: ochratoxin A; DON: deoxynivalenol; Type B: any total of deoxynivalenol, 3-acetyl-deoxynivalenol, 15-acetyl-deoxynivalenol, deoxynivalenol-3-glucoside, nivalenol, or fusarenon-X; T2: T-2 toxin; FUM: fumonisins including B1, B2, B3; ZEA: zearalenone; FA: fusaric acid; ^2^ Pigs/trt: number of pigs per treatment; ^3^ Ini. Wt., kg: initial weight of pigs at the start of the trial. Trials not reporting initial weight are marked with a “-”; ^4^ Phase: pig growth phase reported by each trial of either (N) nursery/immature or (GF) growing or finishing; ^5^ Days: number of days over which the trial was conducted; ^6^ YCWE, kg/t: yeast cell wall extract (Mycosorb^®^, Alltech, Inc.) inclusion rate during mycotoxin challenges. Multiple treatment inclusion rates are separated by “,”; ^7^ Contam.: source of mycotoxin contamination used for the mycotoxin challenge diets. Sources include naturally contaminated feedstuffs, mycotoxins derived from culture material, pure crystalline mycotoxins, or a mixture of different sources; ^8^ Cat.1/Cat. 2: category 1 or category 2. Meta-regression category 1 represents trials with reported mycotoxin levels at or below EU and US regulatory advisory levels, while category 2 represents trials with at least one reported mycotoxin above regulatory advisory limits.

**Table 2:** Overall mean effect size estimates from the random-effect meta-analysis for feeding control non-mycotoxin contaminated diets, mycotoxin treatments, or yeast cell wall extract treatment during mycotoxin challenges on the performance of growing pigs.

					Heterogeneity Test		
Item ^1^	No. Comp.^2^	Mean Effect Size	95% CI ^3^	*p*-Value	*I*^2^ (%) ^4^	*p*-Value	Eggar *p*-Value ^5^
ADG, g/d ^6^							
MT-CTRL	20	−84.0	−105.3, −62.8	<0.001	66.81	0.0003	0.4427
YCWE+MT-MT	31	17.0	−1.2, 35.2	0.0679	57.66	0.0003	0.3246
YCWE+MT-CTRL	21	−75.8	−110.8, −40.8	<0.0001	88.85	0.0001	0.7579
ADFI, g/d ^6^							
MT-CTRL	17	−165.0	−215.9, −114.1	<0.0001	69.69	0.0002	0.9190
YCWE+MT-MT	26	18.2	−6.4, 42.7	0.1472	25.94	0.2600	0.3014
YCWE+MT-CTRL	18	−143.8	−202.3, −85.2	<0.0001	79.21	<0.0001	0.6692
G:F ^6^							
MT-CTRL	19	0.0008	−0.0316, 0.0332	0.9627	92.33	<0.0001	0.1543
YCWE+MT-MT	30	0.0026	−0.0091, 0.0142	0.6666	70.42	<0.0001	0.4022
YCWE+MT-CTRL	20	−0.0051	−0.0320, 0.0217	0.7071	89.48	<0.0001	0.1138

^1^ Treatments represent CTRL: control diets reported to contain undetectable or minimal mycotoxin contamination; MT: diets with reported mycotoxin contamination; and YCWE+MT: yeast cell wall extract (Mycosorb^®^, Alltech, Inc.) diets containing both YCWE and mycotoxins. Effects were determined by the differences between treatments of MT and control (MT-CTRL), YCWE+MT and MT (YCWE+MT-MT), or YCWE+MT and control (YCWE+MT-CTRL); ^2^ No. Comp.: number of different trial comparisons available for each treatment and performance variable; ^3^ 95% CI: 95% confidence interval; ^4^ *I*^2^: percentage of between-study variation; ^5^ Eggar *p*-Value: Eggar test for asymmetry for gauging publication bias; ^6^ ADG: average daily gain; ADFI: average daily feed intake; G:F: gain to feed ratio.

**Table 3:** Mycotoxin regulatory guidance levels utilized as a reference for the determination of meta-regression categories 1 and 2. Limits are based on those suggested for finished feed for swine [5,6,7,83].

	Guidance Levels (μg/kg)	
Mycotoxin	Immature	Finishing
Aflatoxins	5	20
Ochratoxin A	50	50
Deoxynivalenol	1000	1000
T-2 + HT-2 toxins	250	250
Fumonisin B1 + B2	5000	5000
Zearalenone	100	250

## Supplementary Materials

The following supporting information can be downloaded at: https://www.mdpi.com/article/10.3390/toxins15100596/s1, Figure S1: The PRISMA flow diagram showing search, screening and final selection of trials included in the random-effects meta-analysis with meta-regression investigating the influence of mycotoxins with or without supplementation of yeast cell wall extract (Mycosorb®, Alltech Inc.) on growing pigs; Figure S2: Forest plots showing the mean difference in average daily gain for growing pigs from the overall random-effects meta-analysis; Figure S3: Forest plots showing the mean difference in average daily feed intake for growing pigs from the overall random-effects meta-analysis; Figure S4: Forest plots showing the mean difference in gain to feed ratio for growing pigs from the overall random-effects meta-analysis; Figure S5: Funnel plots for assessment of publication bias of studies included in the random-effects meta-analysis for average daily gain; Figure S6: Funnel plots for assessment of publication bias of studies included in the random-effects meta-analysis for average daily feed intake; Figure S7: Funnel plots for assessment of publication bias of studies included in the random-effects meta-analysis for gain to feed ratio.
