# Supplementary material for: Use of Yeast Cell Wall Extract for Growing Pigs Consuming Feed Contaminated with Mycotoxins below or above Regulatory Guidelines: A Meta-Analysis with Meta-Regression
Source: Toxins (Basel). 2023 Oct 3;15(10):596. doi: 10.3390/toxins15100596 (PMC10611179; doi:10.3390/toxins15100596)
Supplement: Supplementary file 1 [file toxins-15-00596-s001.zip › toxins-2586930-supplementary.pdf]

Use of yeast cell wall extract for growing pigs consuming feed contaminated with mycotoxins below or above regulatory guidelines: a meta-analysis with meta-regression.

SUPPLEMENTARY MATERIAL

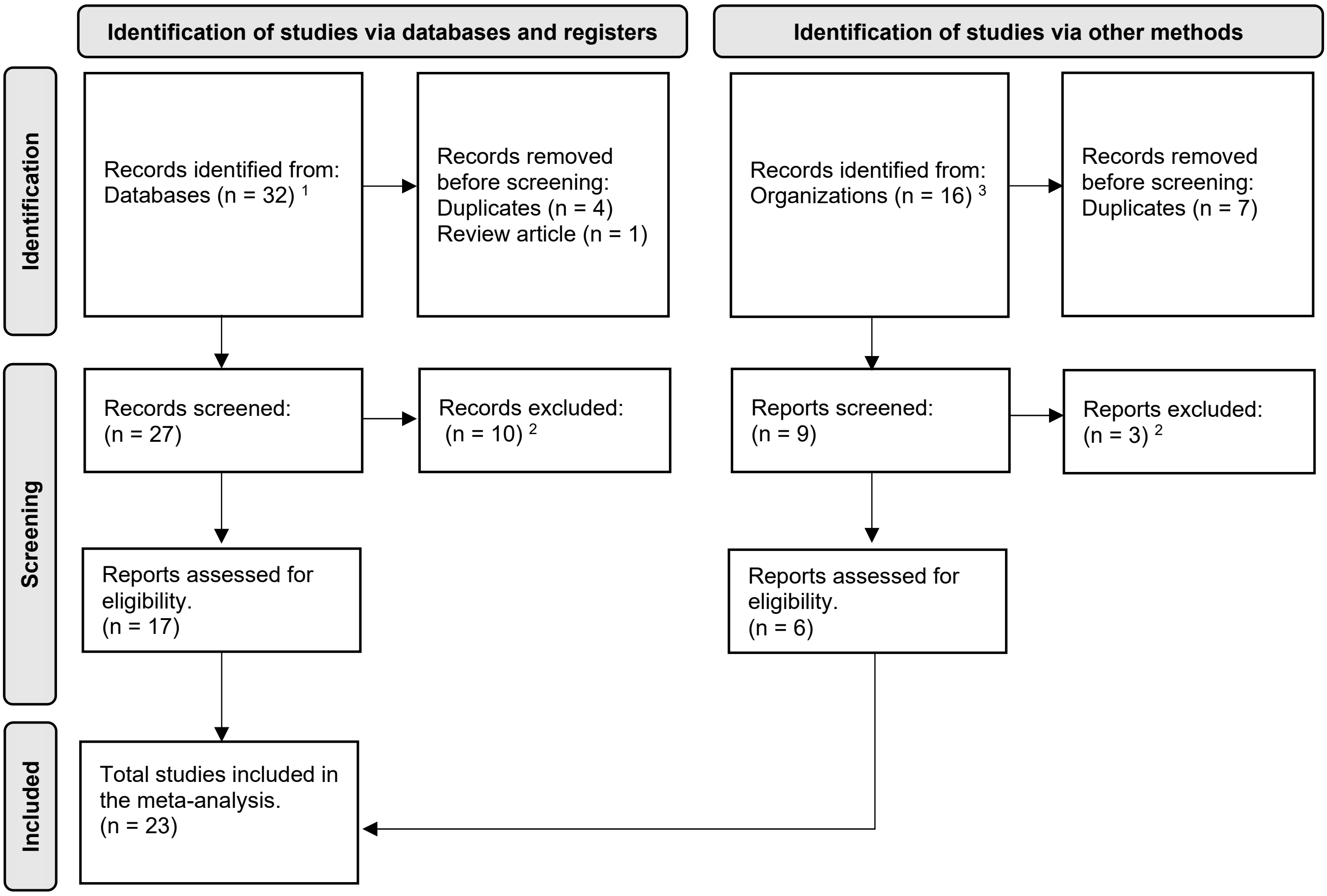

Supplementary Figure S1. The PRISMA flow diagram showing search, screening and final selection of trials included in the random-effects meta-analysis with meta-regression investigating the influence of mycotoxins with or without supplementation of yeast cell wall extract (Mycosorb®, Alltech Inc.) on growing pigs [82]. Search of references was conducted in electronic databases (Google Scholar, Agricola, PubMed), and with keywords that included "mycotoxins" and "pigs" or "swine" in all searches, and at least one of "yeast cell wall extract," "esterified glucomannan," "glucomannan polymer," "Mycosorb®," or "Alltech." Reasons for exclusion were: 1) lack of mycotoxin results reported, 2) growth performance results not measured or reported, 3) incorrect age of pigs, i.e. sows, and 4) product description not clear if product was Mycosorb®. Organizational data was obtained from Alltech's internal database.

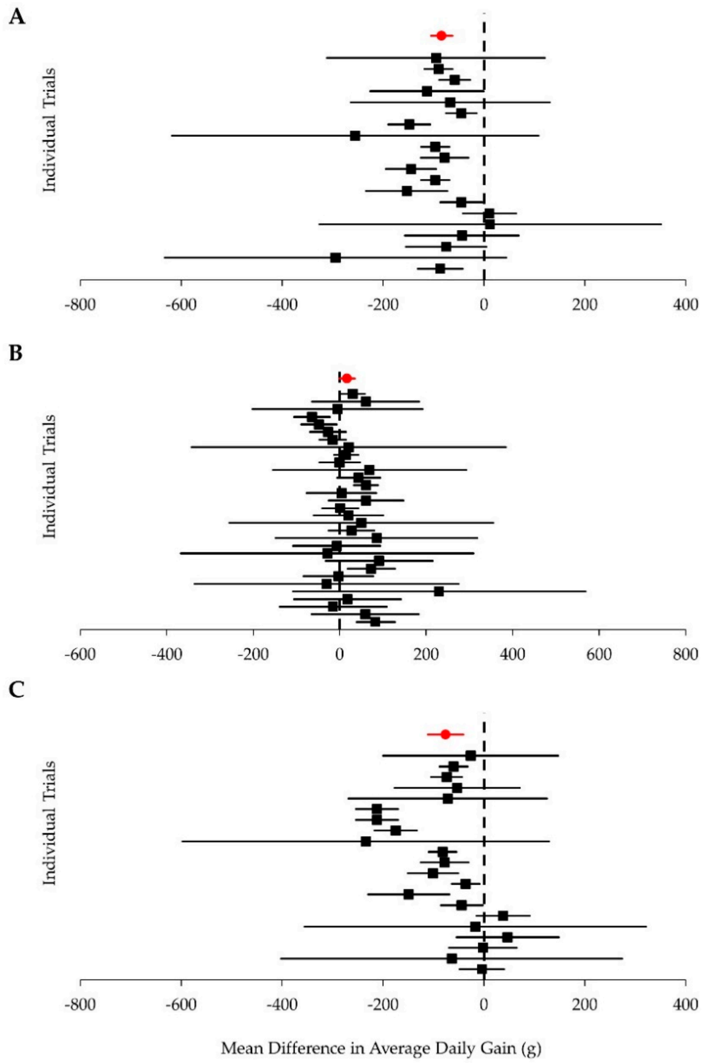

Supplementary Figure S2. Forest plots of the mean differences in average daily gain (g) for pigs from the random-effects meta-analysis. The mean effect sizes with 95% confidence intervals between treatments are shown for individual studies (in black) and the overall difference (in red) for comparisons of pigs fed (**A**) mycotoxins alone (MT) and control without mycotoxins (CTRL), (**B**) yeast cell wall extract during mycotoxin challenges (YCWE+MT, Mycosorb®, Alltech, Inc.) and MT, and (**C**) YCWE+MT and CTRL.

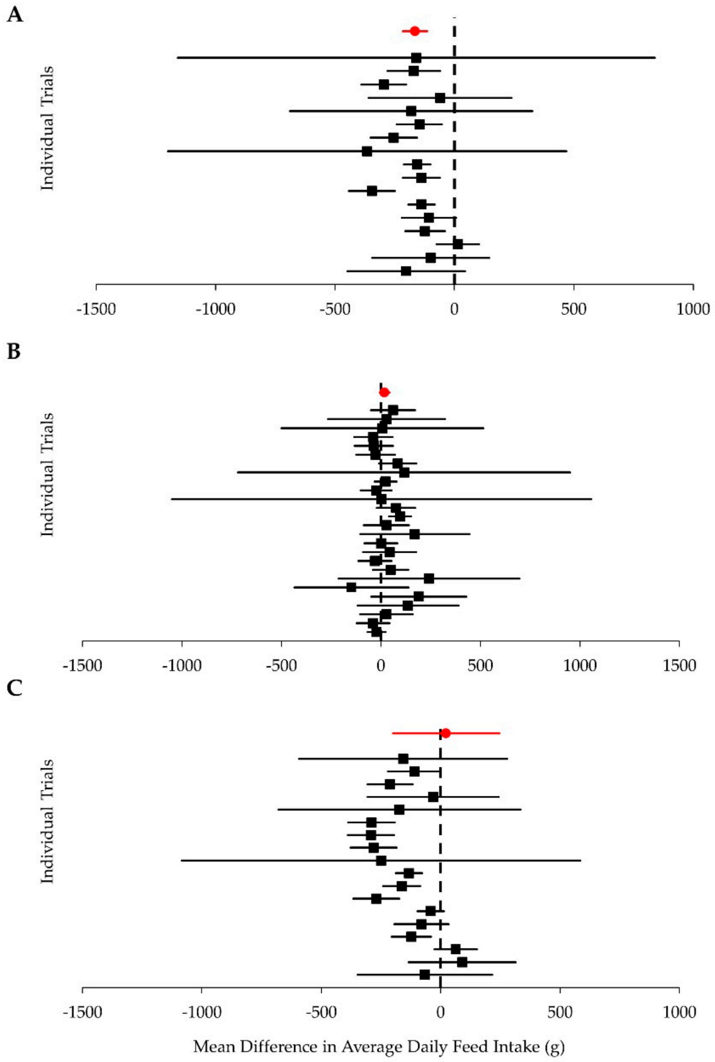

Supplementary Figure S3. Forest plots of the mean differences in average daily feed intake (g) for pigs from the random-effects meta-analysis. The mean effect sizes with 95% confidence intervals between treatments are shown for individual studies (in black) and the overall difference (in red) for comparisons of pigs fed (**A**) mycotoxins alone (MT) and control without mycotoxins (CTRL), (**B**) yeast cell wall extract during mycotoxin challenges (YCWE+MT, Mycosorb®, Alltech, Inc.) and MT, and (**C**) YCWE+MT and CTRL.

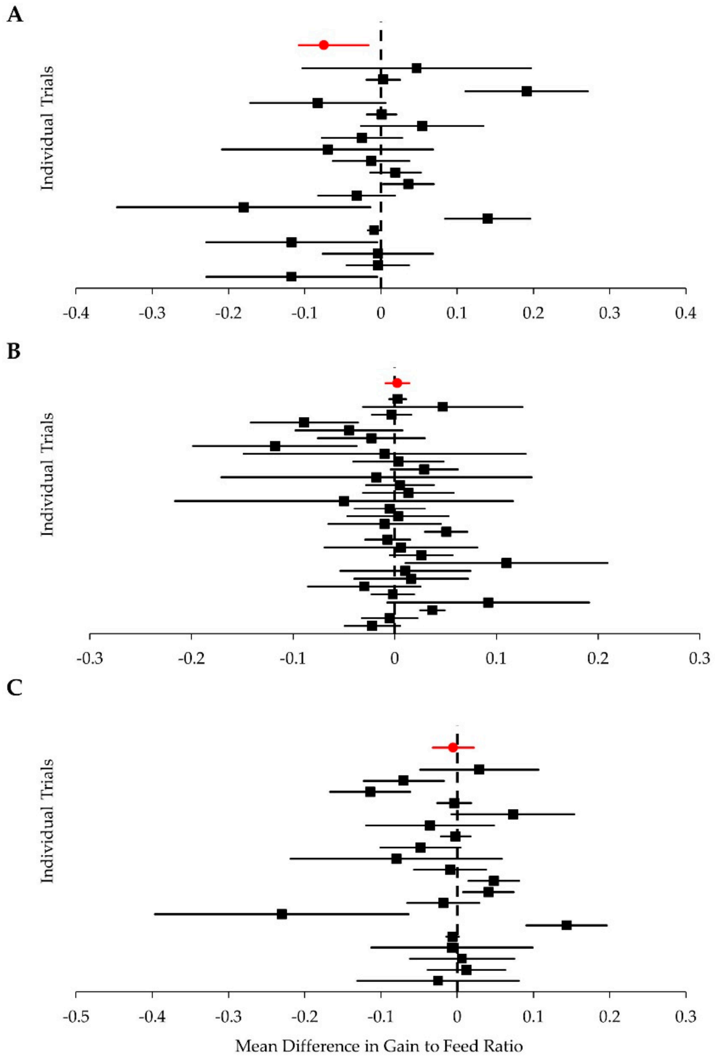

Supplementary Figure S4. Forest plots of the mean differences in gain to feed ratio for pigs from the random-effects meta-analysis. The mean effect sizes with 95% confidence intervals between treatments are shown for individual studies (in black) and the overall difference (in red) for comparisons of pigs fed (**A**) mycotoxins alone (MT) and control without mycotoxins (CTRL), (**B**) yeast cell wall extract during mycotoxin challenges (YCWE+MT, Mycosorb®, Alltech, Inc.) and MT, and (**C**) YCWE+MT and CTRL.

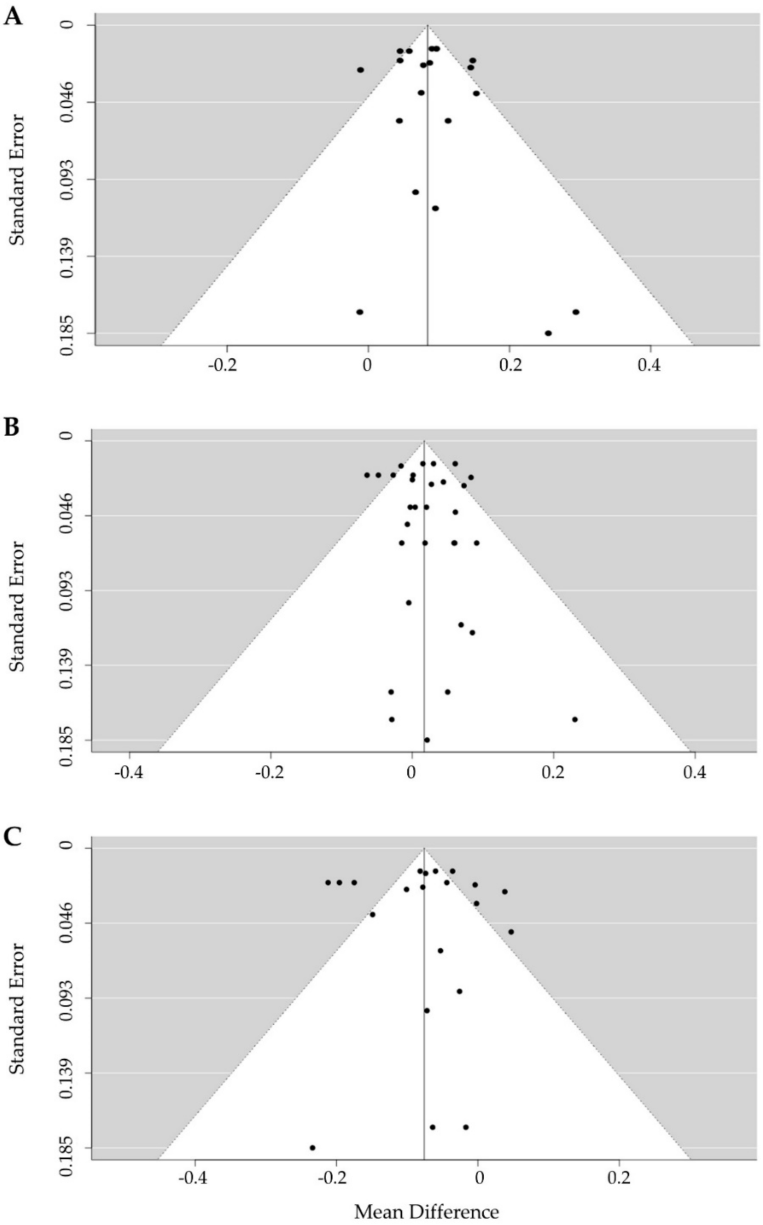

Supplementary Figure S5. Funnel plots for assessment of the publication bias of studies included in the random-effects meta-analysis showing the mean differences in average daily gain (g) of pigs. Circles represent individual study comparisons between treatments of (**A**) mycotoxins alone (MT) and control (CTRL), (**B**) yeast cell wall extract during mycotoxin challenges (YCWE+MT, Mycosorb®, Alltech, Inc.) and MT, and (**C**) YCWE+MT and CTRL.

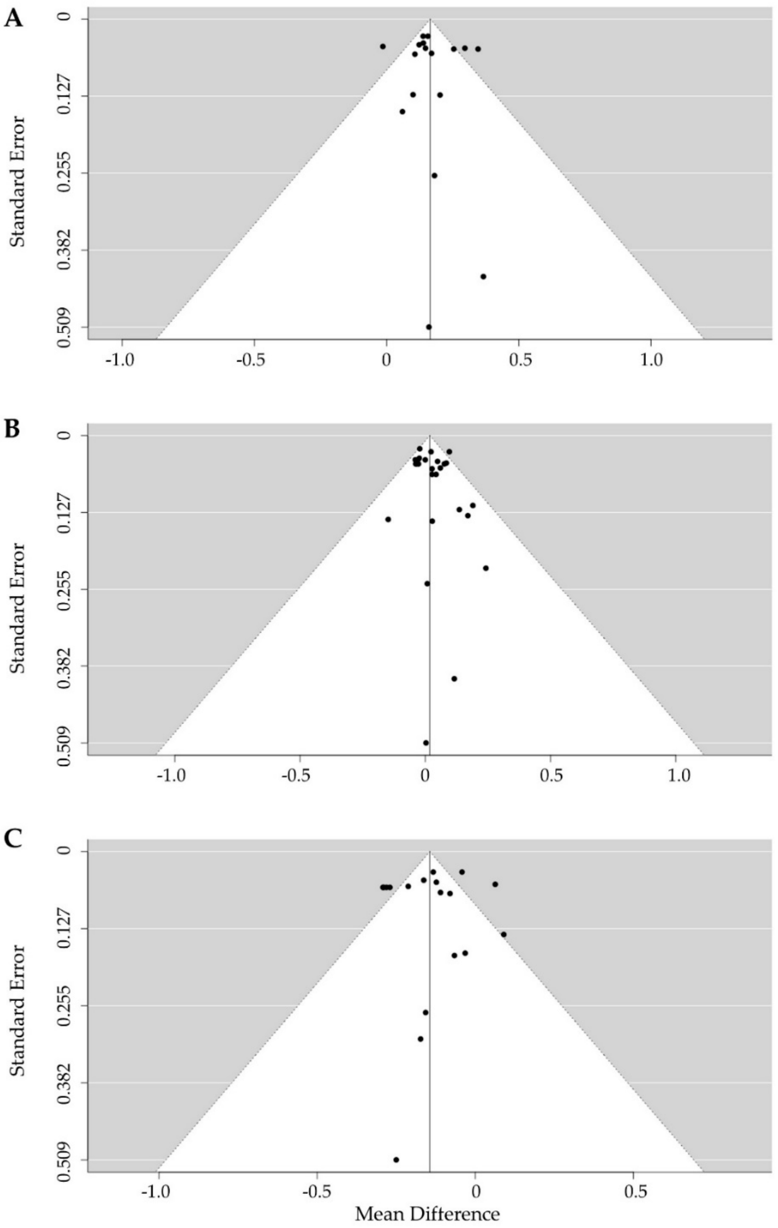

Supplementary Figure S6. Funnel plots for assessment of the publication bias of studies included in the random-effects meta-analysis showing the mean differences in average daily feed intake (g) of pigs. Circles represent individual study comparisons between treatments of (**A**) mycotoxins alone (MT) and control (CTRL), (**B**) yeast cell wall extract during mycotoxin challenges (YCWE+MT, Mycosorb®, Alltech, Inc.) and MT, and (**C**) YCWE+MT and CTRL.

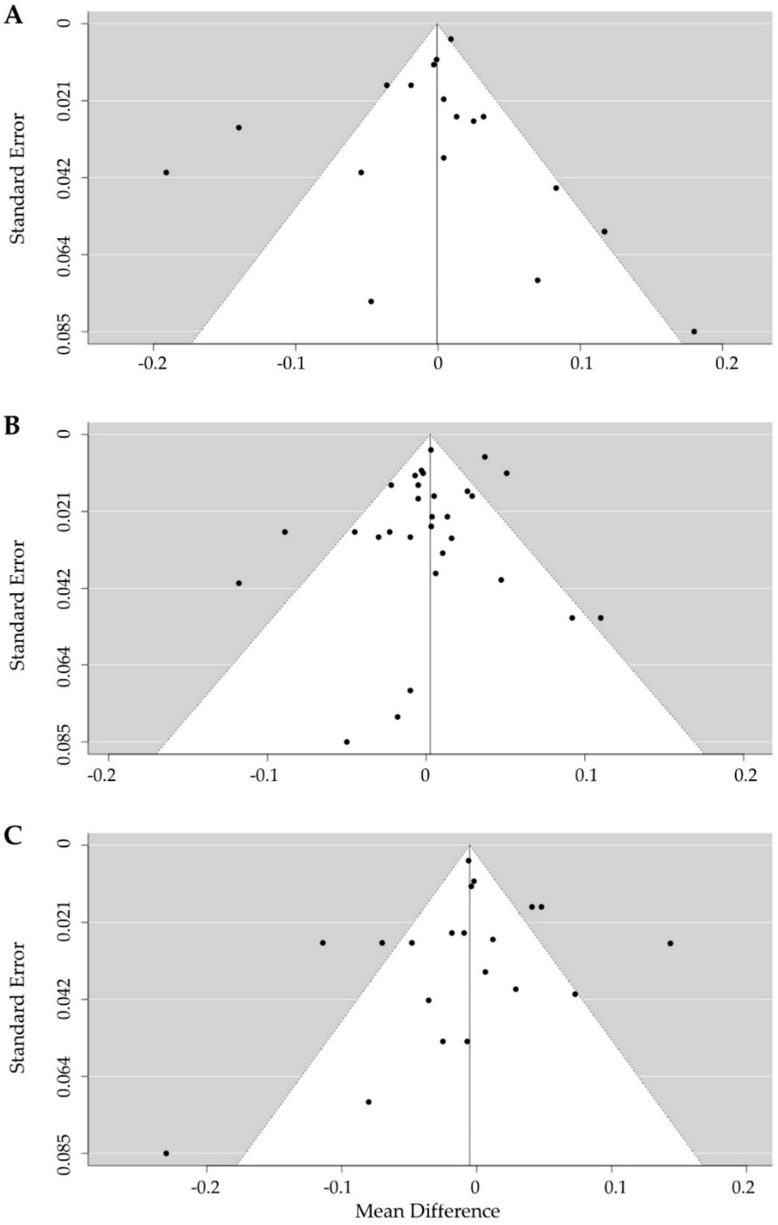

Supplementary Figure S7. Funnel plots for assessment of the publication bias of studies included in the random-effects meta-analysis showing the mean differences in gain to feed ratio of pigs. Circles represent individual study comparisons between treatments of (**A**) mycotoxins alone (MT) and control (CTRL), (**B**) yeast cell wall extract during mycotoxin challenges (YCWE+MT, Mycosorb®, Alltech, Inc.) and MT, and (**C**) YCWE+MT and CTRL.
